# Bioactivity of Methoxylated and Methylated 1-Hydroxynaphthalene-2-Carboxanilides: Comparative Molecular Surface Analysis †

**DOI:** 10.3390/molecules24162991

**Published:** 2019-08-18

**Authors:** Hana Michnová, Šárka Pospíšilová, Tomáš Goněc, Iva Kapustíková, Peter Kollár, Violetta Kozik, Robert Musioł, Izabela Jendrzejewska, Ján Vančo, Zdeněk Trávníček, Alois Čížek, Andrzej Bąk, Josef Jampílek

**Affiliations:** 1 Division of Biologically Active Complexes and Molecular Magnets, Regional Centre of Advanced Technologies and Materials, Faculty of Science, Palacký University, Šlechtitelů 27, 78371 Olomouc, Czech Republic; 2 Department of Infectious Diseases and Microbiology, Faculty of Veterinary Medicine, University of Veterinary and Pharmaceutical Sciences, Palackého třída 1/3, 61242 Brno, Czech Republic; 3 Department of Chemical Drugs, Faculty of Pharmacy, University of Veterinary and Pharmaceutical Sciences, Palackého třída 1/3, 61242 Brno, Czech Republic; 4 Department of Pharmaceutical Chemistry, Faculty of Pharmacy, Comenius University, Odbojárov 10, 83232 Bratislava, Slovakia; 5 Department of Human Pharmacology and Toxicology, Faculty of Pharmacy, University of Veterinary and Pharmaceutical Sciences, Palackého třída 1/3, 61242 Brno, Czech Republic; 6 Institute of Chemistry, University of Silesia, Szkolna 9, 40007 Katowice, Poland; 7 Department of Analytical Chemistry, Faculty of Natural Sciences, Comenius University, Ilkovičova 6, 84215 Bratislava, Slovakia

**Keywords:** hydroxynaphthalenecarboxamides, lipophilicity, X-Ray structure, antistaphylococcal activity, antimycobacterial activity, MTT assay, cytotoxicity, PET inhibition, 3D-QSAR, CoMSA

## Abstract

A series of twenty-six methoxylated and methylated *N*-aryl-1-hydroxynaphthalene- 2-carboxanilides was prepared and characterized as potential anti-invasive agents. The molecular structure of *N*-(2,5-dimethylphenyl)-1-hydroxynaphthalene-2-carboxamide as a model compound was determined by single-crystal X-ray diffraction. All the analysed compounds were tested against the reference strain *Staphylococcus aureus* and three clinical isolates of methicillin-resistant *S.* *aureus* as well as against *Mycobacterium tuberculosis* and *M. kansasii*. In addition, the inhibitory profile of photosynthetic electron transport in spinach (*Spinacia oleracea* L.) chloroplasts was specified. In vitro cytotoxicity of the most effective compounds was tested on the human monocytic leukaemia THP-1 cell line. The activities of *N*-(3,5-dimethylphenyl)-, *N*-(3-fluoro-5-methoxy-phenyl)- and *N*-(3,5-dimethoxyphenyl)-1-hydroxynaphthalene-2-carbox- amide were comparable with or even better than the commonly used standards ampicillin and isoniazid. All promising compounds did not show any cytotoxic effect at the concentration >30 µM. Moreover, an in silico evaluation of clogP features was performed for the entire set of the carboxamides using a range of software lipophilicity predictors, and cross-comparison with the experimentally determined lipophilicity (log *k*), in consensus lipophilicity estimation, was conducted as well. Principal component analysis was employed to illustrate noticeable variations with respect to the molecular lipophilicity (theoretical/experimental) and rule-of-five violations. Additionally, ligand-oriented studies for the assessment of the three-dimensional quantitative structure–activity relationship profile were carried out with the comparative molecular surface analysis to determine electron and/or steric factors that potentially contribute to the biological activities of the investigated compounds.

## 1. Introduction

Hydroxynaphthalenecarboxanilides demonstrated a spectrum of anti-infectious [1,2,3,4,5,6,7,8] and/or anti-proliferative properties [9,10], as was described recently. These structurally simple compounds, regarded as ring analogues of salicylanilides (e.g., niclosamide [11,12,13]), can be considered a part of the group of privileged structures. Their activity is dependent on the mutual position of the carboxamide and the phenolic moieties [1,2,3,5,8]; therefore, analogues with various positions of the phenolic motif show a spectrum of various biological activities. The carboxamide fragment (‑CONH–) mimicking a peptide bond is crucial for the activity, because by means of this moiety a compound is bound to its targets. In terms of the polypharmacology idea, these compounds can be classified as multi-target compounds, since they are able to affect various target structures, especially in microbial pathogens, similarly as salicylanilides [14,15,16,17]. It is assumed that the anilide part of the molecule is responsible for influencing the physicochemical properties and binding strength of the tested compounds to the potential target.

Drug design is a complex issue that is focused on the optimization of the molecular interactions between a drug molecule and a biomolecular target. The contacted drugs or, more generally, ligands (in Latin ‘ligare’ means ‘tie’) and macromolecules initiate the signals that control the substantial effects of living organisms [18]. A description of the intensity of the ligand-target contacts and their interactions require a specific representation for the proper evaluation of quantitative structure–activity relationships (QSAR). Roughly speaking, properties and/or descriptors are two representations of chemical compounds [19]. While properties are measured, descriptors are calculated or, more accurately, are final results of a logic or mathematical procedure transforming the chemical information encoded within symbolic representations of molecules into useful numbers [20]. In this context, even a simple property vs. descriptor paradigm gets fuzzy even when a simple molecular feature such as molecular lipophilicity is analysed. Obviously, lipophilicity can be measured using various experimental approaches that are basically time– and/or material–consuming and require a high purity of the solute; therefore, alternative lipophilicity descriptors have been proposed using mainly in silico predictive models [21]. On the other hand, some methods for the theoretical calculation of lipophilicity might be more or less suitable for specific/heterogeneous series of compounds analysed, thus a variety of approaches should be employed with the results subsequently compared with the empirical data [22]. The quantitative evaluation of the lipophilic characteristics is indispensable at the stage of the generation of ADMET-tailored structure–activity relationships (SAR) models of potential drug molecules. The concepts such as drug-likeness or Lipinski’s rule-of-five (Ro5) highlight the molecular features that are frequently related with the term ‘property’ in drug discovery. Basically, the aforementioned pre-filters are expected to provide a higher success ratio at the advanced development stages based on the ‘fail-early fail-cheaply’ paradigm. Precisely speaking, these filters cannot be solely based on the measurable properties, because in many cases the molecules that are being designed are not accessible for empirical surveys. In consequence, the molecular descriptors need to be calculated in order to foresee the desired substance property. Calculated lipophilicity (clogP) is the most obvious example, since a regression model is built based on ca. 3 × 10^4^ lipophilic measurements [23]. Probably, the property vs. descriptor ‘play’ can be better explained when we realize that actual logP measurement is relatively rarely conducted even if a compound is synthesized.

In the light of the above-mentioned facts, a new series methoxylated and methylated *N*-aryl-1-hydroxynaphthalene-2-carboxanilides was designed and investigated for their physicochemical and biological properties. The compounds were tested for their antistaphylococcal and antimycobacterial activities. Additionally, their ability to inhibit photosynthetic electron transport (PET) in spinach (*Spinacia oleracea* L.) chloroplasts was evaluated, because it was found previously that antimycobacterial activity correlates with PET inhibition profile [1,2,3,4,5,6,7,14,24,25,26,27]. The amide moiety is present in many herbicides acting as PET inhibitors in photosystem (PS) II [28]. The formation of hydrogen bonds between the CONH moiety and the target proteins in photosynthetic centres of thylakoid membranes changes protein conformation resulting in PET inhibition. Amides were described as PS II inhibitors, where they cause the displacement of plastoquinone (Q_B_) from its binding pocket in one of the protein subunits of PS II, the D1 protein [29,30,31].

In addition, another objective of the presented study was the in silico deduction of clogP features for the set of *N*-(methoxy/methyl-phenyl)-1-hydroxynaphthalene-2-carboxamides using a range of software lipophilicity predictors and cross-comparison with experimental lipophilicity (log *k*) in consensus lipophilicity evaluation. Moreover, principal component analysis (PCA) was employed to illustrate noticeable variations with respect to the molecular lipophilicity (theoretical/experimental) and rule-of-five violations. Ligand-oriented studies for the assessment of the three-dimensional (3D)-QSAR profile were performed with comparative molecular surface analysis (CoMSA) to determine the electron and/or steric factors that potentially contribute to the biological activities of the investigated compounds. In fact, probability-guided CoMSA modelling based on the iterative variable elimination-partial least squares method (IVE-PLS) facilitated the visualization of the pharmacophore patterns that are potentially valid for the activity of the analysed carboxanilides.

## 2. Results and Discussion

### 2.1. Chemistry

**Scheme 1:**
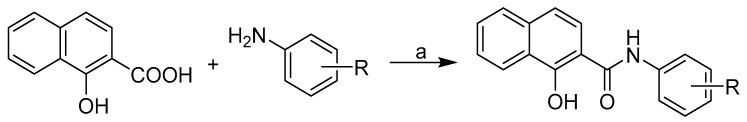
Synthesis of ring-substituted 1-hydroxynaphthalene-2-carboxanilides **1**–**26**. Reagents and conditions: (a) PCl_3_, chlorobenzene, MW, 15 min, [1,10].

The reaction of 1-hydroxynaphthalene-2-carboxylic acid and an appropriate substituted aniline with phosphorus trichloride in dry chlorobenzene under microwave conditions gave a series of methoxylated and methylated *N*-aryl-1-hydroxynaphthalene-2-carboxanilides **1**–**26**, see Scheme 1 and Table 1. Compounds **1**–**4**, **8**–**10** [1], **5**–**7**, **11**–**19**, **21**, **22**, and **24**–**26** [10] were published recently.

### 2.2. X-ray Crystallography

**Figure 1:**
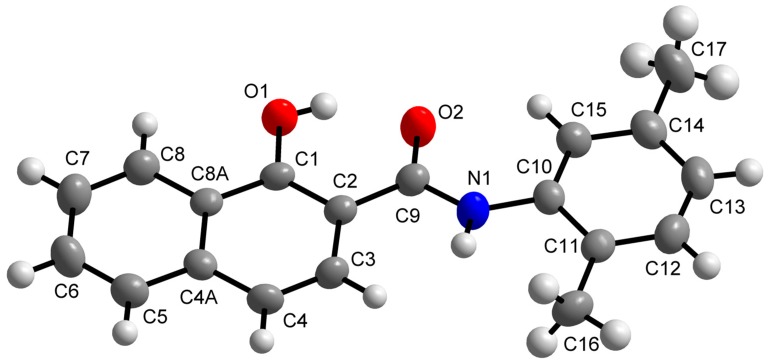
Molecular structure of *N*-(2,5-dimethylphenyl)-1-hydroxynaphthalene-2-carboxamide (**11**). Non-H atoms are drawn as thermal ellipsoids at 50% probability level. H-atoms are not labelled and are displayed as balls in light-grey colour.

The molecular structure of *N*-(2,5-dimethylphenyl)-1-hydroxynaphthalene-2-carboxamide (**11**) is shown in Figure 1, while its crystal data and structure refinement are given in Table 2. The selected bond lengths and angles are summarized in Table S1 in Supplementary Materials. This compound showed in vitro antistaphylococcal activities and could be obtained in the form of single crystals suitable for X-ray structure determination. The molecular structure of compound **11** consists of individual molecules which are stabilized by intramolecular O–H···O hydrogen bonds (Figure S1, Table S2 in Supplementary Materials). Moreover, the C–H···O and C–H···C non-covalent interactions connect the individual molecules into a 3D supramolecular structure. The molecule of **11** is nearly planar. The least-square plane fitted through all the non-H atoms of **11** revealed that the maximal deviation from the plane has been found for the N1 atom (0.1022(23) Å). The molecule contains naphthalene and benzene rings that are nearly coplanar with the angle of 3.014(59)°. The bond lengths and angles of compound **11** are of typical values and comparable with those in already published structures involving the *N*-(phenyl)-naphthalene-2-carboxamide moiety, except for the C9=O2 distance (1.249(3) Å), which is slightly influenced by the presence of the O1–H1A···O2, C5‑H5A···O2 and C15–H15A···O2 non-covalent contacts. To date, 31 structures with the mentioned moiety have been deposited within the Cambridge Structural Database (CSD) [32] revealing the values of median C=O and N–C(O) bond lengths of 1.227(17) Å and 1.358(32) Å, respectively.

### 2.3. In Silico clogP Estimation and Experimental Lipophilicity Specification

**Figure 2:**
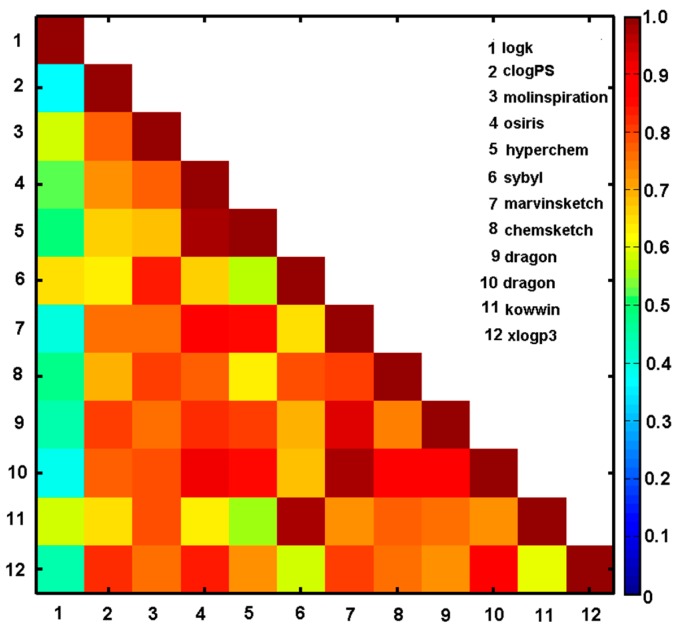
Matrix of correlation coefficients of linear relationships between experimental lipophilicity (log*k*^1^) and calculated lipophilicity for series **1**–**26**.

The estimation of the lipophilic values for the ensemble of *N*-(methoxy/methyl-phenyl)-1-hydroxynaphthalene-2-carboxamides was performed using a range of in silico logP predictors, for instance, clogPS, Molinspirations, OSIRIS, HyperChem 7.0, Sybyl X, MarvinSketch 15, ACD/ChemSketch 2015, Dragon6.0, Kowwin, and XlogP3. The numerical values of the theoretically estimated partition coefficients and the empirically specified log *k* parameters are listed in Table 1 and Table 3. The corresponding logP estimators deduced by the set of alternative methods were (inter-)correlated with each other and cross-compared with the experimental values as shown in Figure 2 and Table S3 (Supplementary Materials). Unfortunately, the majority of the applied programs (apart from clogPS, Molinspirations, Sybyl X, ChemSketch, Kowwin) do not differentiate the calculated lipophilicity for positional isomers (see Table 3). Not surprisingly, some observed variations in clogP values are probably the consequence of different in silico principles (descriptor/atom/fragment-based) implemented in the software and/or models applied (training datasets) [33]. Generally, a pretty high inter-correlation (ranging from r = 0.56 to r = 0.98) within the estimated values of logP was noticed for the analysed group of compounds as illustrated by the triangular matrix of linear correlation parameters in Figure 2. On the other hand, relatively poor correlation between the empirical lipophilicity (log *k*) and theoretical logP estimators was recorded with the best match depicted for Sybyl-X (r = 0.65) and Molinspiration (r = 0.59) software. Strangely enough, the higher inter-correlation between calculated clogP values, the worse cross-correlation with the experimental data. Obviously, models are as good as the training data applied at the modelling stage; therefore, the poor predictive logP performance can be partially elucidated by insufficient coverage of the structural space by measured compounds.

The IVE-PLS procedure applied for the overall clogP matrix (X_26 × 11_) with the log *k* parameter as a dependent variable selected Molinspiration, OSIRIS, Sybyl-X, ChemSketch, and MarvinSketch software as significant contributors to the final model. Noticeably, not only the best inter-correlated logP estimators were indicated, but also some balanced selection that prevents data overfitting was observed by an evenly covering broader range of theoretical procedures. Subsequently, the averaged values of the specified lipophilicity estimators were correlated with the experimental data (log *k*) with the correlation coefficient r = 0.57 in the consensus clogP procedure.

### 2.4. Variable-Oriented Similarity Evaluation

**Figure 3:**
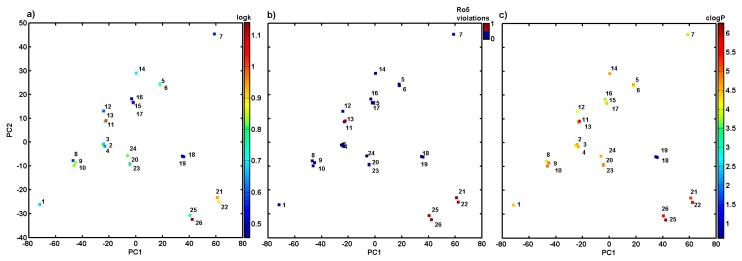
Projection of derivatives **1**–**26** on plane defined by first vs. second principal component with: log *k* (**a**), number of Ro5 rule violations (**b**) and calculated in Sybyl-X lipophilicity (**c**). Colours code values of experimental lipophilicity, Ro5 violations and theoretical lipophilicity.

The descriptor-based similarity assessment was conducted using the PCA method on the pool of variables derived from Dragon 6.0 software for the set of the investigated molecules. The initial ensemble of the selected parameters (4885) was confined to 2733 variables because all columns with nearly constant (standard deviation <10^−4^) or constant values and with missing values have been excluded at the pre-processing stage. The resulting dataset was arranged into a matrix X with 26 rows representing objects (molecules) and 2733 columns representing numerical variables (descriptors). Significant variations of the examined set of carboxamide derivatives regarding their structure and lipophilic profiles were visualized using centred and standardized data since the library of input parameters contains variables of different orders of magnitude. The determination of the model complexity expressed by the amount of principal components (PCs) is based on the percentage of the modelled variance. In fact, PCA model with first four PCs described 79.50% of the total data variance, while the first two PCs accounted for 61.91%. Not surprisingly, the examined analogues can be classified into groups taking into account structural parameters –positional isomers are generally grouped together as illustrated in Figure 3a. Obviously, the major structural variations of the remaining ones from all were indicated for unsubstituted compound **1** (only hydrogen as an R substituent within the phenyl ring) and tri-substituted compound **7**. A noticeable separation of di-substituted compounds with a CF_3_ moiety (**21**, **22** and **25**, **26**) was observed as well. Moreover, the indicated compounds violate Ro5 as shown in Figure 3b. A detailed examination of the theoretical lipophilicity (calculated in Sybyl-X) revealed that CF_3_ containing compounds are characterized with higher values of clogP estimators (clogP > 5) and the experimental log *k* property as depicted in Figure 3a,c, respectively. On the other hand, ‘a good drug-like score does not make a molecule a drug’ [34], but in the case of the specified compounds, Ro5 violation can partially explain the observed decline in the activities.

### 2.5. Biological Screening

As mentioned above, the biological activities of all the compounds were tested against *S. aureus* ATCC 29213 as the reference and quality control strain and three clinical isolates of methicillin-resistant *Staphylococcus aureus* (MRSA) [16,17]. For the reduction of risks, the evaluation of the antimycobacterial activity of the compounds was performed against avirulent strain *M. tuberculosis* ATCC 25177/H37Ra that has a similar pathology as *M. tuberculosis* strains infecting humans. This model pathogen is commonly used in basic laboratory screening and represents a good model for testing antitubercular agents [35]. *M. kansasii* DSM 44162 was used as the second strain representing non-tuberculous mycobacteria. They cause, among other diseases, nontuberculous mycobacterial lung infections, which are very common nowadays and can be indistinguishable from tuberculosis [36]. In addition, all the compounds were investigated in relation to the inhibition of photosynthetic electron transport (PET) in spinach (*Spinacia oleracea* L.) chloroplasts. Antimicrobial activities were expressed as minimum inhibitory concentrations (MICs), while PET inhibiting activity as an IC_50_ value (compound concentration causing 50% inhibition of PET). All the results are listed in Table 1. Also, MTT (3-(4,5-dimethylthiazol-2-yl)-2,5-diphenyl- tetrazolium bromide) assay of the most effective antistaphylococcal and antitubercular compounds was conducted, and preliminary in vitro cytotoxicity screening of the most effective antimicrobial compounds was performed using the human monocytic leukemia THP-1 cell line. The cytotoxicity was evaluated as an LD_50_ value (LD_50_—lethal dose to 50% of the cell population).

#### 2.5.1. In Vitro Antistaphylococcal Susceptibility Testing

Only several compounds showed high potency against *S. aureus* ATCC 29213 as well as three clinical MRSA strains. Especially *N*-(3-fluoro-5-methoxyphenyl)-1-hydroxynaphthalene-2-carbox- amide (**23**) and *N*-(3,5-dimethylphenyl)-1-hydroxynaphthalene-2-carboxamide (**13**) showed excellent activity against the reference (MICs = 1.60 and 3.43 µM, respectively) and the MRSA strains (MICs = 3.21 and 6.85 µM, respectively) comparable with or better than that of ampicillin. The potency revealed by compounds **6** (R = 3,5-OCH_3_) and **11** (R = 2,5-CH_3_) against all four staphylococcal strains was also high (MICs = 12.3/24.7 and 13.4/27.5 µM, respectively). Since the MICs of these compounds are very similar, it can be speculated about the specific effectivity against *Staphylococcus* sp. In addition, a standard MTT assay was performed on the most effective compounds. The MTT test can be used to assess cell growth by measuring respiration. The MTT measured viability of bacterial cells less than 70% after exposure to the MIC values for each tested compound is considered as a positive result of this assay. This low level of cell viability indicates inhibition of cell growth by inhibition of respiration [37,38]. It can be concluded the both selected compounds, i.e., **13** (R = 3,5-CH_3_, 19%) and **23** (R = 3-F-5-OCH_3_, 49%), showed significantly less than 70% viability of *S. aureus* ATCC 29213 at the tested concentration equal to MICs (i.e., 3.43 µM (1 µg/mL) and 1.60 µM (0.5 µg/mL), respectively). Due to the limited number of effective compounds, SAR cannot be clearly defined; however, it seems that the activity increases with lipophilicity.

#### 2.5.2. In Vitro Antimycobacterial Testing

**Figure 4:**
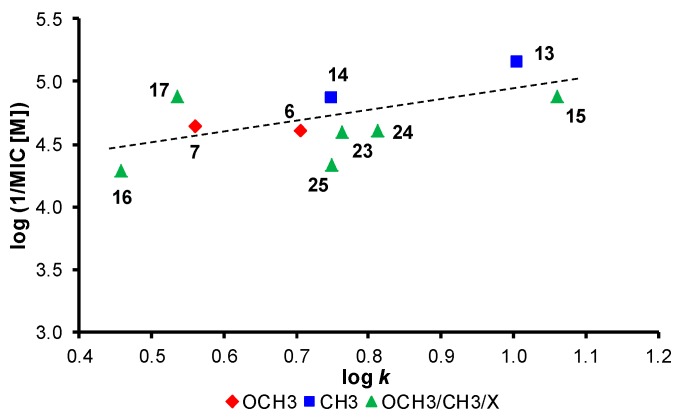
Dependence of antimycobacterial activity of tested compounds against *M. tuberculosis* expressed as log(1/MIC [M]) on lipophilicity expressed as log *k* after elimination of non-active compounds.

Approximately half of the investigated compounds showed activity against both mycobacterial strains. Compound (**13**, R = 3,5-CH_3_) demonstrated the highest activity against *M. tuberculosis* (MIC = 6.86 µM), while compound **23** showed the highest effectivity against *M. kansasii* (MIC = 12.0 µM). Also compounds **14** (R = 2,4,6-CH_3_), **15** (R = 2-MeO-5-Me), **17** (R = 2-CH_3_-5-OCH_3_), **7** (R = 3,4,5-OCH_3_), **24** (R = 2-Cl-5-OCH_3_), **6** (R = 3,5-OCH_3_) and **23** (R = 3-F-5-OCH_3_) expressed high potency against *M. tuberculosis* in the range from 13.0 to 25.6 µM, and compounds **1** (R = H [1]), **5** (R = 2,5-OCH_3_), **6** (R = 3,5-OCH_3_), **13** (R = 3,5-CH_3_) and **9** (R = 3-CH_3_ [1]) showed high potency against *M. kansasii* in the range from 15.2 to 28.8 µM. In addition, an MTT assay was performed with chosen compounds against *M. tuberculosis* H37Ra. Both selected compounds **13** (R = 3,5-CH_3_, 32%) and **26** (R = 3-F-5-OCH_3_, 38%) demonstrated a significant decrease of the viability of *M. tuberculosis* H37Ra at the tested MIC concentrations (i.e., 6.86 µM (2 µg/mL) and 25.6 µM (8 µg/mL), respectively), which means that the compounds have potential to inhibit mycobacterial cell growth through respiratory inhibition [37,39]. The antimycobacterial activity of the compounds against *M. tuberculosis* was expressed as log(1/MIC), and its dependence on lipophilicity expressed as log *k* is illustrated in Figure 4. When inactive compounds are eliminated, in general, it can be stated that the activity slightly increases with increasing lipophilicity. A similar dependence was also found for *M. kansasii*; therefore, it is not shown.

#### 2.5.3. In Vitro Cytotoxicity Assay

The preliminary in vitro cytotoxicity screening of selected compounds **1** (R = H), **6** (R = 3,5-OCH_3_), **11** (R = 2,5-CH_3_), **13** (R = 3,5-CH_3_) and **23** (R = 3-F-5-OCH_3_) was performed using the human monocytic leukaemia THP-1 cells, see Table 1, and the toxicity of the compounds (LD_50_) was compared to that of oxaliplatin (LD_50_ = 1.7 ± 0.6 µM) and camptothecin (0.16 ± 0.07 µM). An agent is considered cytotoxic when it expresses a toxic effect on cells at concentrations up to 10 μM [40]; thus, 30 µM was used as the highest concentration. It can be stated that even the treatment with 30 μM did not lead to any significant toxic affect, i.e., LD_50_ > 30 µM. This observation is in a good agreement with the previous result of antiproliferative assay published recently by Spaczynska et al. [10].

#### 2.5.4. Inhibition of Photosynthetic Electron Transport (PET) in Spinach Chloroplasts

The values of the PET inhibition activity of the evaluated carboxanilides ranged from 8.19 to 569 µM, see Table 1. Compounds **13** (R = 3,5-CH_3_) and **23** (R = 3-F-5-OCH_3_) expressed the highest PET-inhibiting activity (IC_50_ = 8.19 and 10.1 µM, respectively). With respect to this wide range of activities, the trend of increasing PET activity with the increasing lipophilicity within a methoxylated subset of compounds **3**, **4** and **6** and a methylated subset of compounds **8**–**13** (correlation coefficient r = 0.9569, n = 6) can be found, while the dependence of PET activity on log *k* within the combination of substituents (compounds **15**–**26**) is not so clear.

Additionally, we analysed ligand efficiency (LE) and ligand efficiency dependent lipophilicity (LELP) for PET IC_50_ activities (see Table S4 in Supplementary Materials), because ‘lipophilicity alone accounts only partially for the experimental efficacy‘. The widely accepted lower limit of LE is 0.3 [41]. Generally, less active compounds (PET IC_50_ >200) are characterized by lower values of LE <0.25. The desired level for LELP is between 0 and 7.5; however, sometimes the range of −10 to 10 is suggested as well [42]. In fact, all compounds in the analysed series **1**–**26** meet the LELP criterion. Despite LELP has become a useful parameter to follow in the hit→lead optimization, it could not distinguish between ‘lean molecular mass’ and groups of real molecular bulkiness [41].

Based on the structural analogy of the investigated compounds and the presence of the amide bond, it can be hypothesized that these compounds inhibit electron transport in PS II at the site of Q_B_ plastoquinone, i.e., at the acceptor side of PS II similarly as previously published compounds, such as ring-substituted salicylanilides and carbamoylphenylcarbamates [14,24,30], ring-substituted hydroxynaphthalene-2-carboxanilides [1,31], *N*-alkoxyphenylhydroxynaphthalenecarboxamides [27], 8-hydroxyquinoline-2-carboxamides [26], and *N*-substituted 2-aminobenzothiazoles [43]. It is important to underline, as mentioned above and previously [1,3,27,30], that really antimicrobially effective compounds **6** (R = 3,5-OCH_3_), **13** (R = 3,5-CH_3_) and **23** (R = 3-F-5-OCH_3_) were also the most potent PET inhibitors, see Table 1.

### 2.6. Probability-Oriented Pharmacophore Modelling

**Figure 5:**
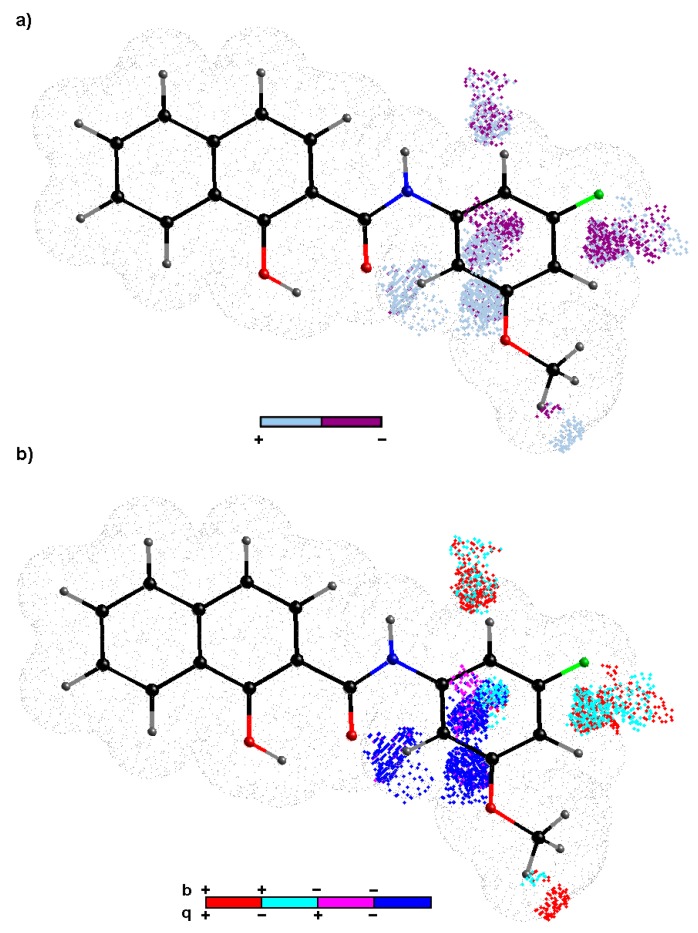
Spatial sectors with greatest contribution into activity specified as CoMSA IVE-PLS for selected 18/8 training/test set samplings. Colours indicate their effect (**a**) and four possible combination of mean charge q and correlation coefficient b values (**b**). Reference compound **23** is plotted as the most active one.

Ligand-oriented studies for in silico assessment of the 3D-QSAR profile were performed with the CoMSA to determine electron and/or steric factors that potentially contribute to the biological activities (expressed in a logarithmic scale) of the investigated compounds [44]. Firstly, the $q_{cv}^{2}$ performance for the whole carboxamide dataset **1**–**26** was examined using the CoMSA approach. Secondly, the external validation by splitting the molecular data into training/test subgroups using Kennard-Stone’a algorithm [45] on descriptor-based data (X_26×2733_) was carried out to evaluate the predictive power of the model with the standard deviation of the error of prediction (SDEP), the mean absolute error (MAE) and $q_{test}^{2}$ statistical metrics. Unfortunately, irrespective of the map size (20 × 20 to 50 × 50) the produced $q_{cv}^{2}$/$q_{test}^{2}$ outcomes were not satisfactory from the statistical point of view for the entire set of measured compound activities. This phenomenon can be partially explained by the insufficient variability among the empirically specified activity data—positional isomers are frequently characterized by the same (or similar) response profiles (see Table 1). Relying exclusively on the training/test choice of datasets is not acceptable in the advanced QSAR studies, therefore an additional evaluation called the stochastic model validation (SMV) was employed as a kind of ‘perturbation’ procedure to scrutinize the structure of the data. Thus, we recurrently sampled all pool of the systematically generated training/test subpopulations ($C_{26}^{8}\;$≈ 1.5 × 10^6^) for activities revealing the greatest diversity. Obviously, the resulting $q_{cv}^{2}$ vs. $q_{test}^{2}$ fluctuation pattern depicted the areas where higher modelling ability is accompanied by the areas of lower model predictive power – preferential selection of training objects produces the decline of the predictive abilities, but ‘the great advantage of the QSAR paradigm lies not in the extrapolation’ with an ‘absolute measure’ of predictivity as was stated by Hansch [46]. Furthermore, the 26/8 training/test samples (3:1 ratio) were selected from regions of a fairly high model ability and predictability to produce namely ‘consensus-based’ pharmacophore maps. Basically, an even compound frequency distribution within the test subset population was observed; however, mainly inactive compounds **2** (R = 2-OCH_3_), **8** (R = 2-CH_3_), **10** (R = 4-CH_3_), **14** (R = 2,4,6-CH_3_), **26** (R = 4-CH_3_-3-CF_3_), moderately active compound **11** (R = 2,5-CH_3_) and active compound **13** (R = 3,5-CH_3_) outnumber the remaining ones. Roughly speaking, the *ortho/para* substituted carboxamide analogues with electron-donating methyl- or methoxy-substituent(s) were preferentially chosen to the test set. On the selected molecules of the training set, the IVE-PLS procedure was applied to eliminate variables with the lowest stability *abs(mean(b)/std(b))* values [47]. The cumulative sum of the common columns for all of the investigated models was calculated and normalized to the range of <0,1>. The spatial pattern illustrated in Figure 5 was produced with the pre-selected cut-off of 0.6 and further filtering of 60% of the CoMSA descriptors that show a relatively small statistical significance for the modelled activity.

The relative contribution of each variable was weighted by the magnitude of the corresponding regression coefficient *b*, where colours were used to indicate the influence of the selected charge (*q*) descriptors on the activity profile to show not only the areas with a positive and/or negative activity contribution (Figure 5a), but also four possible combinations of the mean regression coefficients and charge values (Figure 5b). The detrimental impact on the investigated activity, mainly due to steric and/or electrostatic factors, is indicated by dark spheres as shown in Figure 5a. Conversely, bright areas illustrate the spatial pattern that was foreseen to be occupied by an atom or substituent to enhance the molecule activity profile. It appeared that *ortho* and/or *para* substituents contribute unfavourably to the activity of the analysed carboxamides, as was suggested by the dark areas nearby the phenyl ring in Figure 5a. It seems that both positively and negatively charged groups at these positions (see Figure 5b) have a detrimental impact on the observed activity of the compound. More or less, the same tendency was observed among the positional isomers with *ortho* or *para* ‑OCH_3_ or –CH_3_ group substitution in the phenyl ring (see the experimental data in Table 1). On the other hand, the obtained findings demonstrate the significance of *meta* position, where the increase in the bulkiness appears to be a favourable structural variation as shown in Figure 5a. In fact, the presence of negatively charged motifs seem to contribute favourably (negative regression coefficients) to the observed activity response of the investigated compounds as illustrated in Figure 5b. It corresponds quite well to the enormous enhancement of the activity of the compounds that was observed for *meta* methoxy-based analogues (especially at position 5 of the phenyl ring), for instance, compounds **6** (R = 3,5-OCH_3_) and **23** (R = 3-F-5-OCH_3_). Moreover, the side chain elongation at position 5 with positively charged atom or group may contribute favourably to the activity as depicted in Figure 5b.

In fact, probability-guided 3D-QSAR (CoMSA) modelling based on the iterative variable elimination facilitated the visualization of the pharmacophore patterns that are potentially valid for the activity of the analysed compounds.

## 3. Experimental Section

### 3.1. Chemistry—General Information

#### 3.1.1. General Information

All reagents were purchased from Merck (Darmstadt, Germany) and Fisher Scientific (Waltham, MA, USA). Reactions were performed using a StartSYNTH microwave lab station (Milestone, Sorisole BG, Italy). Melting points were determined on a Kofler hot-plate apparatus (HMK Franz Kustner Nacht BG, Dresden, Germany) and are uncorrected. Infrared (IR) spectra were recorded on a Smart MIRacle ATR ZnSe for Nicolet Impact 410 FT-IR spectrometer (Thermo Scientific, West Palm Beach, FL, USA). The spectra were obtained by the accumulation of 64 scans with 2 cm^−1^ resolution in the region of 4000–650 cm^−1^. All ^1^H- and ^13^C-NMR spectra were recorded on a JEOL ECZR 400 MHz NMR spectrometer (400 MHz for ^1^H and 100 MHz for ^13^C, Jeol, Tokyo, Japan) in dimethyl sulfoxide-*d_6_* (DMSO-*d_6_*). ^1^H and ^13^C chemical shifts (δ) are reported in ppm. High-resolution mass spectra were measured using a high-performance liquid chromatograph Dionex UltiMate^®®^ 3000 (Thermo Scientific) coupled with an LTQ Orbitrap XL^TM^ Hybrid Ion Trap-Orbitrap Fourier Transform Mass Spectrometer (Thermo Scientific) equipped with a HESI II (heated electrospray ionization) source in the positive mode.

#### 3.1.2. Synthesis

General procedure: 1-Hydroxynaphthalene-2-carboxylic acid (5.3 mmol), and the corresponding substituted aniline (5.3 mmol), were suspended in 30 mL of dry chlorobenzene. Phosphorous trichloride (2.65 mmol) was added dropwise, and the reacting mixture was heated using infrared flask-surface control of temperature at maximal allowed power 500 W and 130 °C in the microwave reactor for 15 min. The solvent was evaporated under reduced pressure. Subsequently, the solid residue was washed with 2 M HCl, and the crude product was recrystallized from aqueous ethanol. All the studied compounds are presented in Table 1.

Described anilides **1**–**4**, **8**–**10** were characterized recently by Gonec et al. [1] and **5**–**7**, **11**–**19**, **21**, **22**, **24**–**26** by Spaczynska et al. [10].

*N-(5-Bromo-2-methoxyphenyl)-1-hydroxynaphthalene-2-carboxamide* (**20**). Yield 80%; Mp 157–159 °C; IR (cm^−1^): 3429, 1626, 1599, 1589, 1576, 1530, 1502, 1481, 1409, 1388, 1331, 1273, 1243, 1206, 1174, 1141, 1125, 1087, 1029, 943, 866, 802, 784, 755, 720; ^1^H-NMR (DMSO-*d_6_*), δ: 13.43 (br. s, 1H), 10.49 (s, 1H), 8.33 (d, *J* = 8.2 Hz, 1H), 8.02–8.08 (m, 2H), 7.92 (d, *J* = 8.2 Hz, 1H), 7.67 (t, *J* = 7.3 Hz, 1H), 7.57–7.62 (m, 1H), 7.48 (d, *J* = 8.7 Hz, 1H), 7.41 (dd, *J* = 8.7, 1.8 Hz, 1H), 7.12 (d, *J* = 8.7 Hz, 1H), 3.88 (s, 3H); ^13^C-NMR (DMSO-*d_6_*), δ: 168.05, 158.20, 151.21, 136.08, 129.02, 128.68, 127.66, 127.61, 127.13, 126.01, 124.88, 123.81, 123.16, 118.65, 113.53, 111.32, 109.16, 56.20; HR-MS: [M + H]^+^ calculated 372.02298 m/z, found 372.02444 m/z.

*N-(3-Fluoro-5-methoxyphenyl)-1-hydroxynaphthalene-2-carboxamide* (**23**). Yield 54%; Mp 95–98 °C; IR (cm^−1^): 3439, 1639, 1600, 1592, 1575, 1538, 1468, 1448, 1410, 1388, 1348, 1323, 1276, 1249, 1197, 1174, 1142, 1060, 950, 824, 806, 757, 724, 676; ^1^H-NMR (DMSO-*d_6_*), δ: 13.66 (br. s, 1H), 10.51 (s, 1H), 8.34 (d, 1H, *J* = 8.2 Hz), 8.08 (d, 1H, *J* = 8.8 Hz), 7.92 (d, 1H, *J* = 8.2 Hz), 7.69 (ddd, 1H, *J* = 8.0, *J* = 6.8, *J* = 1.3 Hz), 7.59 (ddd, 1H, *J* = 8.3, *J* = 7.0, *J* = 1.2 Hz), 7.48 (d, 1H, *J* = 8.8 Hz), 7.34 (dt, 1H, *J* = 11.0, *J* = 1.2 Hz), 7.25 (t, 1H, *J* = 1.2 Hz), 6.67 (dt, 1H, *J* = 11.0, *J* = 1.2 Hz), 3.80 (s, 3H); ^13^C-NMR (DMSO-*d_6_*), δ: 169.62, 162.90 (d, *J* = 222.9 Hz), 160.43, 159.90, 139.81 (d, *J* = 13.7 Hz), 136.05, 129.28, 127.50, 126.02, 124.61, 123.09, 122.98, 117.93, 107.551, 103.36 (d, *J* = 1.4 Hz), 100.81 (d, *J* = 26.7 Hz), 97.69 (d, *J* = 24.5 Hz), 55.64; HR-MS: [M + H]^+^ calculated 312.10305 *m/z*, found 312.10295 *m/z*.

#### 3.1.3. Lipophilicity Determination by HPLC (Capacity Factor *k*/Calculated log *k*)

The lipophilicity determination was performed as described by Kapustikova et al. [48]. The log *k* values of individual compounds are shown in Table 1.

#### 3.1.4. X-ray Crystallography

The X-ray data for a colourless crystal of **11** was obtained at 120 K using a Bruker D8 QUEST diffractometer equipped with a PHOTON 100 CMOS detector, using MoK_α_ radiation (λ = 0.71073 Å). The data collection and reduction were realized using the APEX3 software package [49]. The structure of **11** was determined using direct methods (SHELXS) and refined by a full-matrix least-squares procedure (SHELXL) [50]. All hydrogen atoms were found in the difference Fourier maps and refined using a riding model with C–H = 0.95 Å for (CH)_aromatic_ and 0.98 Å for (CH_3_), and with U_iso_(H) = 1.2 U_eq_(CH), and 1.5 U_eq_(CH_3_), respectively. The N–H and O–H hydrogen atoms were refined in a similar manner using AFIX 43 and AFIX 83 instructions, respectively. The graphics were drawn and additional structural calculations were performed by the DIAMOND [51] software. The description of molecular and crystal structures is provided in Figure 2 and Figure S1, respectively, while crystal data and structure refinement, selected bond lengths and angles and selected non-covalent interatomic contacts can be found in Table 2, Tables S1 and S2, respectively.

Crystallographic data has been deposited with the Cambridge Crystallographic Data Centre under CCDC deposition number 1939550. Copies of this information may be obtained free of charge from the Director, CCDC, 12 Union Road, Cambridge CB2 1EY, UK (fax: +44-1223-336033; e-mail: deposit@ccdc.cam.ac.uk or www: http://www.ccdc.cam.ac.uk).

### 3.2. Biological Testing

#### 3.2.1. In Vitro Antibacterial Evaluation

The synthesized compounds were evaluated for in vitro antibacterial activity against representatives of multidrug-resistant bacteria, clinical isolates of methicillin-resistant *Staphylococcus aureus* (MRSA) 63718, SA 630 and SA 3202 [16,17] that were obtained from the National Institute of Public Health (Prague, Czech Republic). *S. aureus* ATCC 29213 was used as the reference and quality control strain. Ampicillin (Sigma, St. Louis, MO, USA) was used as the standard. The screening was performed as described previously [2,3,4,7,16]. The results are summarized in Table 1.

#### 3.2.2. In Vitro Antimycobacterial Evaluation

The evaluation of the in vitro antimycobacterial activity of the compounds was performed against *Mycobacterium tuberculosis* H37Ra ATCC 25177 and *M. kansasii* DSM 44162 by means of the methodology described recently [e.g., 1,3,4–6,15,43]. Isoniazid (Sigma) was used as the standard. The results are summarized in Table 1.

#### 3.2.3. MTT Assay

Compounds were prepared as previously stated and diluted in MH or MB broth for *S. aureus* to achieve the desired final concentrations 0.5 and 1 µg/mL, respectively, for *S. aureus* ATCC 29213, and 2 and 8 µg/mL, respectively, for *M. tuberculosis* ATCC 25177/H37Ra. *M. tuberculosis* ATCC 25177/H37Ra was suspended in ODAC supplemented MB at 1.0 McFarland and then diluted 1:10, using MB as a diluent. The diluted mycobacteria (50 µL) were added to each well containing the compound to be tested. *S. aureus* bacterial suspension in sterile distilled water at 0.5 McFarland was diluted 1:3. Inocula were added to each well by multi-inoculator. Diluted mycobacteria in broth free from inhibiting compounds were used as the growth control. All compounds were prepared in duplicate. Plates were incubated at 37 °C for 7 days for *M. tuberculosis* and 24 h for *S. aureus*. After the incubation period, 10% well volume of MTT (3-(4,5-dimethylthiazol-2-yl)-2,5-diphenyltetrazolium bromide) reagent (Sigma) was mixed into each well and incubated at 37 °C for 4 h in dark for mycobacteria and 1 h for *S. aureus*. Then 100 µL of 17% sodium dodecyl sulphate in 40% dimethylformamide was added to each well. The plates were read at 570 nm. The absorbance readings from the cells grown in the presence of the tested compounds were compared with uninhibited cell growth to determine the relative percent inhibition. The percent inhibition was determined through the MTT assay. The percent viability is calculated through the comparison of a measured value and that of the uninhibited control: % viability = OD_570E_/OD_570P_ × 100, where OD_570E_ is the reading from the compound-exposed cells, while OD_570P_ is the reading from the uninhibited cells (positive control). Cytotoxic potential is determined by a percent viability of <70% [37,38,39,52].

#### 3.2.4. In Vitro Cytotoxicity Assay

Human monocytic leukaemia THP-1 cells were obtained from the European Collection of Cell Cultures (ECACC, Salisbury, UK; Methods of characterization: DNA Fingerprinting (Multilocus probes) and isoenzyme analysis). Cell toxicity was determined using a Cytotoxicity Detection Kit^PLUS^ Lactate dehydrogenase (LDH) assay kit (Roche Diagnostics, Mannheim, Germany) according to the manufacturer’s instructions, as described previously [1,3,4,43]. The results are summarized in Table 1.

#### 3.2.5. Study of Inhibition of Photosynthetic Electron Transport (PET) in Spinach Chloroplasts

The PET-inhibiting investigation using chloroplasts from spinach (*Spinacia oleracea* L.) was performed as described previously [1,2,3,4,24,30,43]. The results are summarized in Table 1. The comparable IC_50_ value of the selective herbicide 3-(3,4-dichlorophenyl)-1,1-dimethylurea, DCMU (Diuron^®®^, Sigma) as the standard was used. The results are shown in Table 1.

### 3.3. Theoretical Calculations

#### 3.3.1. Molecular Modelling

In order to eliminate any potential data noise, that might have been introduced by pooling the data sets from various sources, activity data were specified in the same laboratory. The set of in vitro activity values (IC_50_) for the group of *N*-(methoxy/methyl-phenyl)-1-hydroxynaphthalene-2-carbox- amides is listed in Table 1. The particular compound’s model was build using the CACTVS/csed molecular editor, while the spatial geometry of the molecules was specified using a CORINA 3D generator. The structural data conversion was performed with the (inter)change file format converter OpenBabel. Sybyl-X 2.0/Certara software package running on an HP workstation with a Debian 6.0 operating system was used to conduct the majority of the modelling simulations. The standard Tripos force field (POWELL conjugate gradient algorithm) with a 0.01 kcal/mol energy gradient convergence criterion and a distant dependent dielectric constant were employed to optimize the initial geometry of each compound (MAXMIN2 module). The partial atomic charges were specified with the Gasteiger–Hückel method implemented in Sybyl-X for the calculation of the electrostatic potential values. One 20-ordered atom trial alignment on molecule **23** was used to cover the whole bonding topology in the maximal common structure (MCS) using the atom FIT method, which is based on matching the positions of the atoms between the corresponding atom pairs.

The SONNIA software was engaged in the CoMSA analysis to simulate 20 × 20 to 50 × 50 SOMs with a winning distance ranging from 0.2 to 2.0 [53,54]. In order to produce a 2D map of the electrostatic potential for the superimposed molecules, a SOM network was employed using the Cartesian coordinates of the molecular surfaces. Following the AA (Active Analogue) approach the most active molecule **23** was selected as a reference structure (template molecule). The output maps were reshaped into a 400- to 2500-element vector that was subsequently transformed by the PLS method implemented in the MATLAB programming environment.

#### 3.3.2. In Silico Lipophilicity Estimation, PCA and PLS analysis

A diverse set of freely or commercially accessible computer programs (see Table 3) can be employed to specify the theoretical partition coefficient, for instance, AlogPS, milogP, ClogP, HyperChem logP, MarvinSketch logP, ChemSketch logP, Dragon AlogP/MlogP, Kowwin and XlogP3.

Principal Component Analysis (PCA) is a linear projection method that condenses a multidimensional data (organised in matrix X_mxn_ with the rows corresponding to *m* objects and the columns corresponding to *n* parameters) into a few explanatory principal components (PCs) [55,56]. PCA is a classic method of data exploration, which allows to reduce data dimensionality, because a restricted ensemble of the orthogonal PCs creates a basis of the lower-dimensional space.

Partial least squares (PLS) is a multivariate statistical method that can analyse the input data with strong co-linearity and model, simultaneously a relationship between response data (dependent variable) Y and an ensemble of descriptors (independent variables) X is expressed briefly in the form of the following formula:

Y = X *b* + e (1)

where *b* is a vector of the regression coefficients and *e* is a vector of errors.

#### 3.3.3. Iterative PLS-based Variable Elimination

A set of descriptors that encode the molecular structure are usually (inter-)correlated significantly, hence the relatively high data uncertainty is problematic in the rehearsal to assign and foreseen a specified property. In fact, redundant descriptors can have a negative impact on the outcome of the regression analysis; therefore, a selection of informative variables might noticeably improve the PLS modelling. Despite the variable elimination/selection is an elaborate issue at least a few chemometric algorithms have been proposed recently [57]. For instance, the iterative variable elimination IVE-PLS that was effectively applied in the multidimensional-QSAR procedures can be regarded as an enhancement of the single-step UVE procedure that was originally proposed by Centner et al. for the specification of the variables to be eliminated [58]. Roughly speaking, the entire algorithm is composed of four basic stages including:

Stage 1. Standard PLS analysis with LOO-CV to evaluate the performance of the PLS model
Stage 2. Elimination of a matrix column with the lowest *abs(mean(b)/std(b))* value
Stage 3. Standard PLS analysis of the new matrix without the column eliminated in stage 2
Stage 4. Recurrent repetition of steps 1–3 to maximize the LOO parameter

## 4. Conclusions

Twenty-six variously methylated and methoxylated *N*-aryl-1-hydroxynaphthalene- 2-carboxanilides were evaluated against *S. aureus*, three clinical methicillin-resistant *S. aureus* isolates, *M. tuberculosis*, and *M. kansasii*. The cytotoxic effects of the chosen effective compounds were estimated on human monocytic leukaemia THP-1 cells using (LDH) assay. All the compounds were tested as inhibitors of photosynthetic electron transport (PET) in spinach chloroplasts. *N*-(3,5-Dimethylphenyl)-1-hydroxynaphthalene-2-carboxamide (**13**), *N*-(3-fluoro-5-methoxyphenyl)- 1-hydroxynaphthalene-2-carboxamide (**23**) and *N*-(3,5-dimethoxy- phenyl)-1-hydroxynaphthalene- 2-carboxamide (**6**) showed comparable with or even better activities than the used standards, such as ampicillin and isoniazid. The MTT test of the most effective compounds against *S. aureus* and *M. tuberculosis* suggested that a respiratory chain of both pathogens might be one of the possible sites of action of these potential antimicrobial multitarget compounds. The effective compounds demonstrated no cytotoxic effects up to the concentration LD_50_ >30 µM; therefore, the tested carboxanilides can be considered as non-toxic agents for subsequent design of novel therapeutic agents. PCA was employed to illustrate noticeable variations with respect to the molecular lipophilicity (theoretical (clogP)/experimental (log *k*)) and Ro5 violations. Moreover, the ligand-oriented studies for the assessment of the 3D-QSAR profile were carried out with the CoMSA to determine the electron and/or steric factors that potentially contribute to the biological activities of the investigated compounds. It seems that both positively and negatively charged groups at *ortho* and/or *meta* positions have a detrimental impact on the observed carboxamide activity. More or less, the same tendency was observed among the positional isomers with *ortho* or *para* –OCH_3_ or –CH_3_ group substitution in the phenyl ring. On the other hand, the obtained findings demonstrate the significance of the *meta* position, where an increase in the bulkiness appears to be a favourable structural variation. In fact, the presence of negatively charged motifs seems to contribute favourably (negative regression coefficients) to the observed activity response of the investigated compounds. It corresponds quite well with the enormous increase of activity of compounds that was observed for the *meta* methoxy-based analogues (especially at position 5 of the phenyl ring), for instance compounds **6** (R = 3,5-OCH_3_) and **23** (R = 3-F-5-OCH_3_).

## Figures and Tables

**Table 1:** Compositions of *N*-substituted 1-hydroxynaphthalene-2-carboxanilides, experimental log *k* values, in vitro antistaphylococcal activities MIC [μM] compared to ampicillin (AMP), in vitro antimycobacterial activities MIC [μM] compared to isoniazid (INH), in vitro cytotoxicity (Tox) assay (LD_50_ [μM]) of chosen compounds and IC_50_ [μM] values related to PET inhibition in spinach chloroplasts compared to 3-(3,4-dichlorophenyl)-1,1-dimethylurea (DCMU) standard.

Comp.	R	log *k*	MIC [μM]						Tox LD_50_ [μM]	PET IC_50_ [μM]
			SA	MRSA 63718	MRSA SA 630	MRSA SA 3202	MT	MK		
**1**	H ^a^	0.6769	>972	>972	243	122	486	**15.2**	>30	31.3
**2**	2-OCH_3_^a^	0.8584	>873	>873	>873	>873	436	>873	–	199
**3**	3-OCH_3_^a^	0.6713	109	>873	>873	>873	436	>873	–	23.5
**4**	4-OCH_3_^a^	0.6284	873	>873	>873	>873	436	>873	–	79.5
**5**	2,5-OCH_3_^b^	0.8712	791	395	395	197	396	24.7	–	201
**6**	3,5-OCH_3_^b^	0.7048	**12.3**	**24.7**	**24.7**	**24.7**	**24.7**	**24.7**	>30	**13.4**
**7**	3,4,5-OCH_3_^b^	0.5603	724	724	724	724	22.6	90.5	–	468
**8**	2-CH_3_^a^	0.5650	115	231	231	231	462	115	–	62.8
**9**	3-CH_3_^a^	0.8235	115	923	923	462	462	28.8	–	20.0
**10**	4-CH_3_^a^	0.8294	923	923	923	923	462	57.7	–	28.7
**11**	2,5-CH_3_^b^	0.7155	**13.7**	**27.5**	**27.5**	**27.5**	110	220	>30	52.4
**12**	2,6-CH_3_^b^	0.5990	55.0	110	110	110	110	220	–	60.3
**13**	3,5-CH_3_^b^	1.0030	**3.43**	**6.85**	**6.85**	**6.85**	**6.86**	**27.4**	>30	**8.19**
**14**	2,4,6-CH_3_^b^	0.7477	>838	>838	>838	>838	13.1	104	–	295
**15**	2-OCH_3_-5-CH_3_^b^	1.0603	>832	>832	>832	>832	13.0	52.0	–	588
**16**	2-OCH_3_-6-CH_3_^b^	0.4574	>832	>832	>832	>832	52.0	104	–	747
**17**	2-CH_3_-5-OCH_3_^b^	0.5362	52.0	832	104	208	13.0	104	–	142
**18**	2-OCH_3_-4-NO_2_^b^	0.5434	756	756	756	756	378	756	–	495
**19**	2-OCH_3_-5-NO_2_^b^	0.4827	756	756	756	756	378	756	–	155
**20**	2-OCH_3_-5-Br	1.0432	687	687	687	687	344	687	–	569
**21**	2-OCH_3_-5-CF_3_^b^	0.9428	708	708	708	708	354	708	–	26.4
**22**	3-CF_3_-4-OCH_3_^b^	0.8915	354	354	354	5.53	354	88.5	–	18.5
**23**	3-F-5-OCH_3_	0.7629	**1.60**	**3.21**	**3.21**	**3.21**	**25.6**	**12.0**	>30	**10.1**
**24**	2-Cl-5-OCH_3_^b^	0.8130	781	781	781	781	24.4	781	–	60.9
**25**	2-CH_3_-5-CF_3_^b^	0.7479	741	741	741	741	46.3	92.6	–	36.7
**26**	3-CF_3_-4-CH_3_^b^	1.1411	741	741	741	741	371	741	–	16.3
**AMP**	–	–	5.72	45.8	45.8	45.8	–	–	–	–
**INH**	–	–	–	–	–	–	36.6	233	–	–
**DCMU**	–	–	–	–	–	–	–	–	–	2.1

^a^ described in [1], ^b^ described in [10], SA *= Staphylococcus aureus* ATCC 29213; MRSA *=* clinical isolates of methicillin-resistant *S. aureus* 63718, SA 630, and SA 3202 (National Institute of Public Health, Prague, Czech Republic); MT = *Mycobacterium tuberculosis* H37Ra ATCC 25177, MK = *M. kansasii* DSM 44162.

**Table 2:** Crystal data and structure refinement for **11**.

Formula	C_19_H_17_NO_2_
Formula weight	291.33
Temperature	120(2) K
Wavelength	0.71073 Å
Crystal system	Monoclinic
Space group	*P*2_1_/n
Unit cell dimensions	*a* = 6.887(4) Å, α = 90°
	*b* = 16.134(11) Å, β = 99.81(2)°
	*c* = 13.620(8) Å, γ = 90°
Volume	1491.3(15) Å^3^
*Z*	4
Density (calculated)	1.298 g/cm^3^
Absorption coefficient	0.084 mm^−1^
F(000)	616
Crystal size	0.240 × 0.200 × 0.180 mm
Theta range for data collection	1.974 to 25.074°
Index ranges	−8 ≤ h ≤ 8, −19 ≤ k ≤ 19, −16 ≤ l ≤16
Reflections collected	14255
Independent reflections	2635 [R(int) = 0.1005]
Completeness to θ	25.074° (99.7%)
Absorption correction	Semi-empirical from equivalents
Refinement method	Full-matrix least-squares on *F*^2^
Data/restraints/parameters	2635/0/201
Goodness-of-fit on *F*^2^	1.065
Final *R* indices [I > 2σ(I)]	*R*_1_ = 0.0586, w*R*_2_ = 0.1246
*R* indices (all data)	*R*_1_ = 0.1246, *wR*_2_ = 0.1506
Largest diff. peak and hole	0.196 and −0.228 e.Å^−3^

**Table 3:** Theoretically estimated partition coefficient calculated by set of alternative methods for *N*-(methoxy/methyl-phenyl)-1-hydroxynaphthalene-2-carboxamides **1**–**26**.

No.	logP ^a^	miLogP ^b^	ClogP ^c^	ClogP ^d^	ClogP ^e^	ClogP ^f^	ClogP ^g^	MlogP ^h^	AlogP ^i^	ClogP ^j^	ClogP ^k^
**1**	3.98	4.27	3.65	3.51	4.43	3.75	4.50	3.91	3.26	4.47	4.20
**2**	4.27	4.28	3.58	3.25	3.93	3.59	4.40	3.61	3.24	3.99	4.17
**3**	4.25	4.30	3.58	3.25	4.52	3.59	4.66	3.61	3.24	4.55	4.17
**4**	4.23	4.33	3.58	3.25	4.52	3.59	4.45	3.61	3.24	4.55	4.17
**5**	3.95	4.31	3.51	3.00	3.96	3.44	4.61	3.06	3.22	4.07	4.15
**6**	4.02	4.31	3.51	3.00	4.55	3.44	4.70	3.06	3.22	4.63	4.15
**7**	3.47	3.90	3.44	2.75	3.81	3.28	4.46	2.51	3.21	3.98	4.12
**8**	4.44	4.67	3.99	3.97	4.29	4.26	4.96	4.15	3.74	4.46	4.57
**9**	4.47	4.69	3.99	3.97	4.94	4.26	4.96	4.15	3.74	5.02	4.57
**10**	4.47	4.72	3.99	3.97	4.94	4.26	4.96	4.15	3.74	5.02	4.57
**11**	4.72	5.09	4.34	4.44	4.79	4.78	5.42	4.38	4.23	5.00	4.93
**12**	4.69	4.13	4.34	4.44	4.14	4.78	5.42	4.38	4.23	4.44	4.93
**13**	4.74	5.09	4.34	4.44	5.44	4.78	5.42	4.38	4.23	5.57	4.93
**14**	4.54	4.53	4.68	4.91	4.64	5.29	5.88	4.61	4.71	4.99	5.30
**15**	4.57	4.70	3.93	3.72	4.43	4.11	4.86	3.84	3.73	4.54	4.54
**16**	4.50	4.68	3.93	3.72	3.78	4.11	4.86	3.84	3.73	3.98	4.54
**17**	4.59	4.70	3.93	3.72	4.37	4.11	5.12	3.84	3.73	4.54	4.54
**18**	4.10	4.21	2.66	1.27	4.21	3.53	4.79	3.39	3.13	4.53	4.00
**19**	4.07	4.21	2.66	1.27	4.21	3.53	4.95	3.39	3.13	4.53	4.00
**20**	4.07	5.06	4.31	4.04	4.99	4.36	5.86	3.95	3.99	4.88	4.87
**21**	4.82	5.15	4.43	4.14	5.23	4.47	6.44	4.18	4.18	4.95	5.06
**22**	4.82	5.15	4.43	4.14	5.82	4.47	6.26	4.18	4.18	5.52	5.06
**23**	4.65	4.42	3.69	3.39	4.86	3.74	5.20	3.73	3.44	4.75	4.27
**24**	5.14	4.93	4.19	3.77	4.58	4.20	5.26	3.84	3.90	4.63	6.08
**25**	5.12	5.54	4.85	4.86	5.63	5.14	6.31	4.99	4.68	5.42	5.45
**26**	5.07	5.54	4.85	4.86	6.28	5.14	6.31	4.99	4.68	5.98	5.45

^a^ clogPS, ^b^ Molinspirations, ^c^ OSIRIS property explorer, ^d^ HyperChem 7.0, ^e^ Sybyl X, ^f^ Marvin Sketch (ChemAxon) 15, ^g^ ChemSketch 2015, ^h^ Dragon 6.0, ^i^ Dragon 6.0, ^j^ Kowwin, ^k^ XlogP3).

## Supplementary Materials

The supplementary material is available online. Figure S1: Part of the crystal structure of N-(2,5-dimethylphenyl)-1-hydroxynaphthalene-2-carboxamide (11), showing O–H···O and C–H···O non-covalent contacts (green dashed lines). Symmetry code: (i) x+1/2,-y+1/2,z-1/2; Table S1: Selected bond lengths (Å) and angles (°) in 11; Table S2: Parameters (in Å, °) of selected non-covalent contacts within crystal structure of 11; Table S3: Matrix of correlation coefficients (n = 26, α = 0.05) of linear relationships between particular partition coefficients and experimental lipophilicity data (log k) for *N*-(methoxy/methyl-phenyl)-1-hydroxynaphthalene-2-carboxamides 1–26; Table S4: Physicochemical properties and calculated HAC, LE and LELP values of 1–26 analogues.
