# Supplementary material for: Bioactivity of Methoxylated and Methylated 1-Hydroxynaphthalene-2-Carboxanilides: Comparative Molecular Surface Analysis
Source: Molecules. 2019 Aug 18;24(16):2991. doi: 10.3390/molecules24162991 (PMC6720605; doi:10.3390/molecules24162991)

checkCIF/PLATON report

Structure factors have been supplied for datablock(s) j

THIS REPORT IS FOR GUIDANCE ONLY. IF USED AS PART OF A REVIEW PROCEDURE FOR PUBLICATION, IT SHOULD NOT REPLACE THE EXPERTISE OF AN EXPERIENCED CRYSTALLOGRAPHIC REFEREE.

No syntax errors found. CIF dictionary Interpreting this report

Datablock: j

```
Bond precision:  C-C = 0.0040 A          Wavelength=0.71073

Cell:             a=6.887(4)     b=16.134(11)      c=13.620(8)
                  alpha=90       beta=99.81(2)     gamma=90
Temperature:      120 K

                Calculated                 Reported
Volume          1491.3(16)                 1491.3(15)
Space group     P 21/n                     P 21/n
Hall group      -P 2yn                     -P 2yn
Moiety formula  C19 H17 N O2               ?
Sum formula     C19 H17 N O2               C19 H17 N O2
Mr              291.34                     291.33
Dx,g cm-3       1.298                      1.298
Z               4                          4
Mu (mm-1)       0.084                      0.084
F000            616.0                      616.0
F000'           616.27
h,k,lmax        8,19,16                    8,19,16
Nref            2643                       2635
Tmin,Tmax       0.980,0.985                0.980,0.985
Tmin'           0.980

Correction method= # Reported T Limits: Tmin=0.980 Tmax=0.985
AbsCorr = MULTI-SCAN

Data completeness= 0.997          Theta(max)= 25.074

R(reflections)= 0.0586( 1487)     wR2(reflections)= 0.1506( 2635)

S = 1.065                 Npar= 201
```

The following ALERTS were generated. Each ALERT has the format
test-name_ALERT_alert-type_alert-level.
Click on the hyperlinks for more details of the test.

Alert level C

```
PLAT414_ALERT_2_C Short Intra D-H..H-X       H1B      ..H3A             1.91 Ang.
                                                      x,y,z  =      1_555 Check
PLAT420_ALERT_2_C D-H Without Acceptor       N1       --H1B      .    Please Check
PLAT906_ALERT_3_C Large K Value in the Analysis of Variance ......     5.976 Check
PLAT911_ALERT_3_C Missing FCF Refl Between Thmin & STh/L=    0.596         9 Report
PLAT978_ALERT_2_C Number C-C Bonds with Positive Residual Density.         0 Info
```

Alert level G

```
PLAT007_ALERT_5_G Number of Unrefined Donor-H Atoms .............          2 Report
PLAT066_ALERT_1_G Predicted and Reported Tmin&Tmax Range Identical         ? Check
PLAT883_ALERT_1_G No Info/Value for _atom_sites_solution_primary .    Please Do !
```

0 **ALERT level A** = Most likely a serious problem - resolve or explain
0 **ALERT level B** = A potentially serious problem, consider carefully
5 **ALERT level C** = Check. Ensure it is not caused by an omission or oversight
3 **ALERT level G** = General information/check it is not something unexpected

2 ALERT type 1 CIF construction/syntax error, inconsistent or missing data
3 ALERT type 2 Indicator that the structure model may be wrong or deficient
2 ALERT type 3 Indicator that the structure quality may be low
0 ALERT type 4 Improvement, methodology, query or suggestion
1 ALERT type 5 Informative message, check

It is advisable to attempt to resolve as many as possible of the alerts in all categories. Often the minor alerts point to easily fixed oversights, errors and omissions in your CIF or refinement strategy, so attention to these fine details can be worthwhile. In order to resolve some of the more serious problems it may be necessary to carry out additional measurements or structure refinements. However, the purpose of your study may justify the reported deviations and the more serious of these should normally be commented upon in the discussion or experimental section of a paper or in the "special_details" fields of the CIF. checkCIF was carefully designed to identify outliers and unusual parameters, but every test has its limitations and alerts that are not important in a particular case may appear. Conversely, the absence of alerts does not guarantee there are no aspects of the results needing attention. It is up to the individual to critically assess their own results and, if necessary, seek expert advice.

Publication of your CIF in IUCr journals

A basic structural check has been run on your CIF. These basic checks will be run on all CIFs submitted for publication in IUCr journals (*Acta Crystallographica*, *Journal of Applied Crystallography*, *Journal of Synchrotron Radiation*); however, if you intend to submit to *Acta Crystallographica Section C* or *E* or *IUCrData*, you should make sure that full publication checks are run on the final version of your CIF prior to submission.

Publication of your CIF in other journals

Please refer to the *Notes for Authors* of the relevant journal for any special instructions relating to CIF submission.

PLATON version of 03/05/2019; check.def file version of 29/04/2019

Datablock j - ellipsoid plot

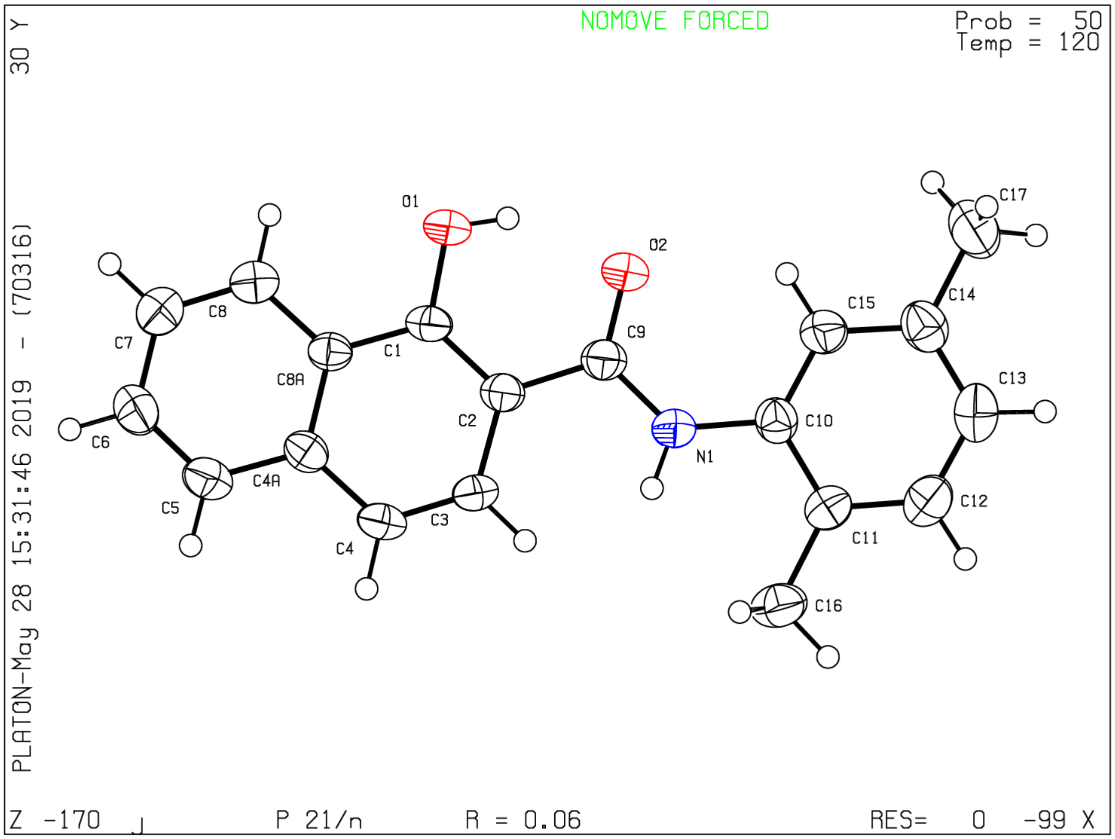

Supplement: Supplementary file 1 [file molecules-24-02991-s001.zip › Supplementary Files/checkcif_Platon_report_basic_deposit_dime.pdf]
