# Supplementary material for: Bioactivity of Methoxylated and Methylated 1-Hydroxynaphthalene-2-Carboxanilides: Comparative Molecular Surface Analysis
Source: Molecules. 2019 Aug 18;24(16):2991. doi: 10.3390/molecules24162991 (PMC6720605; doi:10.3390/molecules24162991)
Supplement: Supplementary file 1 [file molecules-24-02991-s001.zip › Supplementary Files/Supplementary materials-560220.pdf]

Supplementary Materials

Bioactivity of Methoxylated and Methylated 1-Hydroxynaphthalene-2-Carboxanilides: Comparative Molecular Surface Analysis†

Hana Michnová [1,2], Šárka Pospíšilová [1,2], Tomáš Goněc [3,*], Iva Kapustíková [4,*], Peter Kollár [5], Violetta Kozik [6], Robert Musioł [6], Izabela Jendrzejewska [6], Ján Vančo [1], Zdeněk Trávníček [1], Alois Čížek [2], Andrzej Bąk [6,*] and Josef Jampílek [1,7,*]

[1] Division of Biologically Active Complexes and Molecular Magnets, Regional Centre of Advanced Technologies and Materials, Faculty of Science, Palacký University, Šlechtitelů 27, 78371 Olomouc, Czech Republic; michnova.hana@gmail.com (H.M.); sharka.pospisilova@gmail.com (S.P.); jan.vanco@upol.cz (J.V.); zdenek.travnicek@upol.cz (Z.T.)

[2] Department of Infectious Diseases and Microbiology, Faculty of Veterinary Medicine, University of Veterinary and Pharmaceutical Sciences, Palackého třída 1/3, 61242 Brno, Czech Republic; cizeka@vfu.cz

[3] Department of Chemical Drugs, Faculty of Pharmacy, University of Veterinary and Pharmaceutical Sciences, Palackého třída 1/3, 61242 Brno, Czech Republic

[4] Department of Pharmaceutical Chemistry, Faculty of Pharmacy, Comenius University, Odbojárov 10, 83232 Bratislava, Slovakia

[5] Department of Human Pharmacology and Toxicology, Faculty of Pharmacy, University of Veterinary and Pharmaceutical Sciences, Palackého třída 1/3, 61242 Brno, Czech Republic; kollarp@vfu.cz

[6] Institute of Chemistry, University of Silesia, Szkolna 9, 40007 Katowice, Poland; violetta.kozik@us.edu.pl (V.K.); robert.musiol@us.edu.pl (R.M.); izabela.jendrzejewska@us.edu.pl (I.J.)

[7] Department of Analytical Chemistry, Faculty of Natural Sciences, Comenius University, Ilkovičova 6, 84215 Bratislava, Slovakia

* Correspondence: t.gonec@seznam.cz (T.G.); kapustikova@fpharm.uniba.sk (I.K.); andrzej.bak@us.edu.pl (A.B.); josef.jampilek@gmail.com (J.J.)

† Preliminary results presented at the 22nd International Electronic Conference on Synthetic Organic Chemistry, 15 November–15 December 2018; Available Online: https://sciforum.net/conference/ecsoc-22.

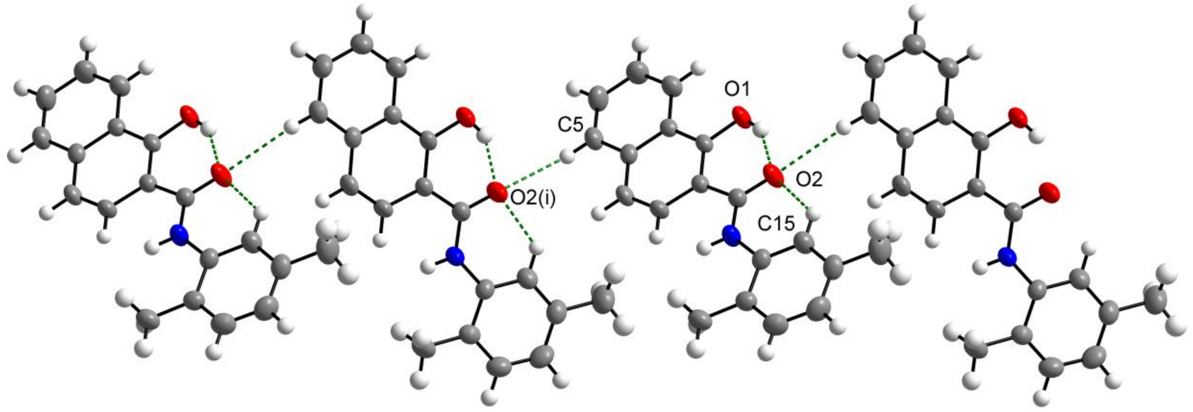

Figure S1. Part of the crystal structure of *N*-(2,5-dimethylphenyl)-1-hydroxynaphthalene-2-carboxamide (**11**), showing O–H···O and C–H···O non-covalent contacts (green dashed lines). Symmetry code: (i) x+1/2,-y+1/2,z-1/2.

Table S1. Selected bond lengths (Å) and angles (°) in **11**.

O(1)-C(1)	1.349(3)
O(2)-C(9)	1.249(3)
N(1)-C(9)	1.351(3)
N(1)-C(10)	1.418(3)
C(1)-C(2)	1.387(4)
C(1)-C(8A)	1.415(4)
C(2)-C(3)	1.429(3)
C(2)-C(9)	1.466(4)
C(9)-N(1)-C(10)	130.9(2)
O(1)-C(1)-C(2)	122.4(2)
O(1)-C(1)-C(8A)	116.6(2)
C(2)-C(1)-C(8A)	121.0(2)
C(1)-C(2)-C(3)	118.0(2)
C(1)-C(2)-C(9)	118.9(2)
C(3)-C(2)-C(9)	123.1(2)
C(4)-C(3)-C(2)	122.3(3)
O(2)-C(9)-N(1)	120.9(3)
O(2)-C(9)-C(2)	121.4(2)
N(1)-C(9)-C(2)	117.7(2)
C(15)-C(10)-C(11)	121.2(3)
C(15)-C(10)-N(1)	122.6(3)
C(11)-C(10)-N(1)	116.2(2)

Table S2. Parameters (in Å, °) of selected non-covalent contacts within crystal structure of **11**.

D-H···A	d(D–H)	d(H···A)	d(D···A)	<(DHA)
O1-H1A...O2	0.84	1.78	2.524(3)	146.8
C15-H15A...O2	0.95	2.23	2.858(4)	122.6
C5-H5A...O2(i)	0.95	2.59	3.429(4)	146.8

Symmetry transformation used to generate equivalent atoms: (i) x+1/2,-y+1/2,z-1/2.

Table S3. Matrix of correlation coefficients (n=26, α=0.05) of linear relationships between particular partition coefficients and experimental lipophilicity data (log *k*) for *N*-(methoxy/methyl-phenyl)-1-hydroxynaphthalene-2-carboxamides **1**–**26**.

	log *k*	logP[a]	miLogP[b]	ClogP[c]	ClogP[d]	ClogP[e]	ClogP[f]	ClogP[g]	MlogP[h]	AlogP[i]	ClogP[j]	ClogP[k]
**log*k***	1											
**logP[a]**	0.37	1										
**miLogP[b]**	0.59	0.78	1									
**ClogP[c]**	0.53	0.73	0.78	1								
**ClogP[d]**	0.49	0.67	0.68	0.97	1							
**ClogP[e]**	0.65	0.64	0.83	0.66	0.57	1						
**ClogP[f]**	0.40	0.76	0.75	0.89	0.85	0.65	1					
**ClogP[g]**	0.47	0.70	0.81	0.77	0.63	0.79	0.81	1				
**MlogP[h]**	0.44	0.81	0.76	0.82	0.80	0.70	0.93	0.74	1			
**AlogP[i]**	0.39	0.77	0.79	0.92	0.85	0.68	0.98	0.87	0.88	1		
**ClogP[j]**	0.58	0.65	0.79	0.64	0.56	0.97	0.72	0.77	0.75	0.73	1	
**ClogP[k]**	0.44	0.82	0.76	0.83	0.73	0.59	0.81	0.76	0.72	0.86	0.60	1

[a] clogPS, [b] Molinspirations, [c] OSIRIS property explorer, [d] HyperChem 7.0, [e] Sybyl X, [f] Marvin Sketch (ChemAxon) 15, [g] ChemSketch 2015, [h] Dragon 6.0, [i] Dragon 6.0, [j] Kowwin, [k] XlogP3.

Table S4. Physicochemical properties and calculated HAC, LE and LELP values of **1**–**26** analogues.

Comp.	R	log *k*	HAC	PET	LE[a]	LELP[a]
**1**	H[a]	0.6769	20	31.3	0.3153	2.1468
**2**	2-OCH_3[a]	0.8584	22	199	0.2355	3.6446
**3**	3-OCH_3[a]	0.6713	22	23.5	0.2946	2.2789
**4**	4-OCH_3[a]	0.6284	22	79.5	0.2609	2.4087
**5**	2,5-OCH_3[b]	0.8712	24	201	0.2156	4.0399
**6**	3,5-OCH_3[b]	0.7048	24	13.4	0.2651	2.6583
**7**	3,4,5-OCH_3[b]	0.5603	26	468	0.1793	3.1250
**8**	2-CH_3[a]	0.5650	21	62.8	0.2801	2.0169
**9**	3-CH_3[a]	0.8235	21	20.0	0.3133	2.6288
**10**	4-CH_3[a]	0.8294	21	28.7	0.3123	2.6560
**11**	2,5-CH_3[b]	0.7155	22	52.4	0.2724	2.6266
**12**	2,6-CH_3[b]	0.5990	22	60.3	0.2685	2.2307
**13**	3,5-CH_3[b]	1.0030	22	8.19	0.3047	3.2920
**14**	2,4,6-CH_3[b]	0.7477	23	295	0.2149	3.4796
**15**	2-OCH_3-5-CH_3[b]	1.0603	23	588	0.2575	4.1174
**16**	2-OCH_3-6-CH_3[b]	0.4574	23	747	0.1903	2.4033
**17**	2-CH_3-5-OCH_3[b]	0.5362	23	142	0.2342	2.2894
**18**	2-OCH_3-4-NO_2[b]	0.5434	25	495	0.1851	2.9357
**19**	2-OCH_3-5-NO_2[b]	0.4827	25	155	0.2133	2.2626
**20**	2-OCH_3-5-Br	1.0432	23	569	0.1975	5.2816
**21**	2-OCH_3-5-CF_3[b]	0.9428	26	26.4	0.2465	3.8243
**22**	3-CF_3-4-OCH_3[b]	0.8915	26	18.5	0.2739	3.2548
**23**	3-F-5-OCH_3	0.7629	23	10.1	0.2966	2.5721
**24**	2-Cl-5-OCH_3[b]	0.8130	23	60.9	0.2566	3.1685
**25**	2-CH_3-5-CF_3[b]	0.7479	25	36.7	0.2484	3.0111
**26**	3-CF_3-4-CH_3[b]	1.1411	25	16.3	0.2798	4.0789

[a] as described by Shultz [1], HAC – heavy atom count, LE – ligand efficiency $(1.4 \times pIC_{50})/HAC$, LELP – ligand efficiency dependent lipophilicity clogP/LE

[1] Shultz, M.D. Setting expectations in molecular optimizations: Strengths and limitations of commonly used composite parameters. *Bioorg. Med. Chem.* **2013**, *23*, 5980–5991.
